# Increase in Properties and Self-Healing Ability of Conductive Butyl Rubber/Epoxidized Natural Rubber Composites by Using Bis(triethoxysilylpropyl)tetrasulfide Coupling Agent

**DOI:** 10.3390/polym15030547

**Published:** 2023-01-20

**Authors:** Piyawadee Luangchuang, Kunakorn Chumnum, Ekwipoo Kalkornsurapranee, Yeampon Nakaramontri

**Affiliations:** 1 Sustainable Polymer & Innovative Composite Materials Research Group, Department of Chemistry, Faculty of Science, King Mongkut’s University of Technology Thonburi, Bangkok 10140, Thailand; 2 Department of Physical Science, Faculty of Science, Prince of Songkla University, Songkhla 10400, Thailand

**Keywords:** natural rubber, self-healing, silane coupling agent, butyl rubber, tire application

## Abstract

Flexible self-healing composite was fabricated based on blending the bromobutyl rubber (BIIR) and epoxide natural rubber (ENR) filled with hybrid fillers of carbon nanotubes (CNT) and carbon black (CB). To achieve self-recoverability, modification of BIIR was carried out through butyl imidazole (IM), and the healing capability was then activated by the addition of bis(triethoxysilylpropyl)tetrasulfide (TESPT), which resulted in good dispersion of CNT/CB in BIIR/ENR blends. The silanization of TESPT and CNT/CB hybrid filler surfaces was confirmed by attenuated total reflection-Fourier transform infrared (ATR-FTIR) spectroscopy. Adding CNT/CB and incorporating TESPT into the composites effectively improved the curing and mechanical properties of the blends in terms of estimated crosslink density and tensile modulus. Further, the self-healing propagation rate was enhanced by the thermal conductivity of fillers and the ion–dipole intermolecular forces between the rubber chains, leading to the highest abrasion resistance and electrical conductivity. Using an environmentally friendly process, the recyclability of the self-healing composites was improved by the re-compression of the samples. With this, the constant conductivity relating to the rearrangement of the CNT/CB network is examined related to the usability of the composites at 0 and 60 °C. The conductive composites filled with a TESPT silane coupling agent present an opportunity for vehicle tires and other self-repairing applications.

## 1. Introduction

Research into elastic materials research has been growing for application to vehicle tires. In particular, the electric vehicle (EV) requires tires with low thickness, high durability, and the proper triangle of abrasion, wet grip, and rolling resistance. Biomimetic researchers have long sought to develop recyclable self-healing materials [1] with longer lifespans. Different self-healing materials have been fabricated and used, from solid concrete to soft elastomeric materials. Using a supramolecular assembly approach, the composite materials developed in this study effectively improved the mechanical and dynamic mechanical properties with reproducibility and ease of processing [2,3,4,5].

Wang et al. have developed new rubber substances with dynamic mechanical crosslinked rubber composites based on elastomeric self-healing. The components of these composites bond together and form long crosslinked chains, mainly through hydrogen and ionic bonds [2]. This molecular system can form hundreds of recoverable chains showing apparent self-healing behavior. However, despite these promising results, questions remain about the long-term stability and potential recovery rate of this interconnected network. The properties of the material under different weather and environmental conditions are essential knowledge. From this perspective, focusing on developing a stabilized rubber with self-healing behavior seemed a reasonable approach.

Currently, several types of natural rubber (NR), epoxidized NR [3,4], and synthetic rubber (SR) have been studied, including styrene–butadiene rubber (SBR) [5], butadiene rubber (BR) [5], and their blending compounds—NR/SBR, SBR/BR, and NR/BR/SBR [6,7,8]—regarding their good rolling properties, which are related to the π–π interaction of the benzene ring in SBR and the excellent elasticity of NR, ENR, and BR [9,10]. However, the physical attraction between the molecules is not sufficient for re-crosslinking the molecular chains of the targeted rubber. Thus, to generate reversible crosslinking rubber chains after it was damaged, a simple approach to converting commercially available rubber using Bromo butyl rubber (BIIR) with extraordinary self-healing properties without using conventional crosslinking agents has been reported [11]. The ability of BIIR to react with various amines (e.g., alkyl imidazole) allows ionic functional groups to be introduced simply [12].

However, it has been found that the reinforcing filler hinders the self-healing propagation of the crosslinked rubber due to the rubber molecules trapped on the filler surfaces. This behavior might be eliminated by improving the dispersion and distribution of the fillers inside the rubber matrix; this has not been studied.

Using a silane coupling agent, bis(triethoxysilylpropyl)tetrasulfide (TESPT), improved filler distribution in the non-polar rubber due to improved morphologies and electrical conductivity has been reported. Nakaramontri et al. have shown that to achieve these properties, the percolation threshold concentration of the composites filled with carbon nanotubes (CNT) and CNT/carbon black (CB) was reduced to approximately 1 phr after the addition of TESPT [13]. This improved the filler–rubber interaction, which reduced the re-agglomeration of fillers after mixing because the induced forces were depleted. Thus, fillers formed a three-dimensional pathway in the rubber matrix, and conductivity improved. However, the effect of TESPT on the self-healing ability of conductive BIIR composites has not been investigated, and it is promising to research the improved healing efficiency of composites because the addition of TESPT to a solution can induce ion movement inside NR composites due to the polarity of the ethoxy groups in the TESPT molecules.

Rubber blending may be required to improve the poor quality of usages of the BIIR-CNT/CB composites. In this case, the high molecular weight rubber of NR is considered. It is well-known that NR has lowest abrasion resistance with low heat build-up and hysteresis. This might make it a good composite with the self-healing BIIR, as these rubbers are compatible, and the filler dispersion would improve synergistically along with the healing ability.

Therefore, in the present work, self-healing modified BIIR using butyl imidazole (IM) was prepared and blended with epoxide natural rubber (ENR) composites. To achieve optimal dispersion and dispersion of CNT/CB, the fillers were combined with the ENR matrix using an internal mixer and a two-roll mill. The BIIR/ENR ratio was fixed at 70:30 [14], and 0.06 g/mL of TESPT was added during the preparation of ENR/CNT-CB to prevent CNT-CB re-agglomeration. This work aimed to improve the self-healing ability of rubber composites with enhanced mechanical and dynamical properties, and improved conductivity, which might benefit tire applications in EV cars.

## 2. Experimental

### 2.1. Materials

Bromo butyl rubber (BIIR) was purchased from ExxonMobil Co., Ltd. (Irving, TX, USA). Epoxide natural rubber (ENR) was purchased from Muang Mai Guthrie Public Co., Ltd. (Surat Thani, Thailand). The multi-wall carbon nanotubes (CNT), grade NC7000, were manufactured from Nanocyl S.A. (Sambreville, Belgium). Carbon black (CB), Vulcan XC72, was purchased from Cabot Corporation (Pampa, TX, USA), the 1-butylimidazole (IM) was received from Merck KGaA. (Darmstadt, Germany), the stearic acid was procured from Imperial Chemical Co. Ltd., (Pathum Thani, Thailand), the zinc oxide (ZnO) and sulfur were manufactured from Global Chemical Co., Ltd. (Samutprakarn, Thailand) and Ajax Chemical Co. Ltd. (Samutprakarn, Thailand), respectively, and the 2,2′-Dithiobis-(benzothiazole) (MBTS) was supplied by Flexsys Inc., Termoli, Italy. In addition, the silane coupling agent, namely bis(triethoxysilylpropyl)tetrasulfide (TESPT), was procured from Bossoftical public Co., Ltd. (Songkla, Thailand).

### 2.2. Preparation of BIIR/NR Composites Filled with Hybrid Filler and Silane

Preparation procedure of the BIIR/ENR composites blends managed the formulation in Table 1. The modified BIIR was performed under the specific condition of 40 °C and 60 rpm of rotor speed using an internal mixer (Brabender VR GmbH & Co. KG, Duisburg, Germany). The BIIR was first added into the internal mixer and masticated for at least 3 min before adding 5 phr of IM at the proper concentration [14]. The mixing operation was performed up to 15 min for producing excess ionic crosslinking. The modified BIIR (M-BIIR) were kept without moisture for at least 2 h and labeled as COMP I. Secondly, in a separate mixture, preparation of ENR composites that reinforced with CNT/CB by using an internal mixer and a two-roll mill (Charoen Tut Co., Ltd., Samutprakarn, Thailand) under condition of 130 °C and 60 rpm of rotor speed were carried out. The ENR was initially masticated in an internal mixer for 2 min before adding the hybrid filler, and the mixing continued for another 6 min. After that, added the TESPT into the compound and mixing was continued for another 5 min. In case of ENR compound without TESPT, the mixing was continued for another 11 min after adding the hybrid filler. The compounds were cooled down before mixing again in an internal mixer under condition of 60 °C and 60 rpm of rotor speed. The compounds were masticated for 1 min before the stearic acid, ZnO, MBTS, and sulfur were added, and the mixing was continued for totally 6 min. The pure ENR was carried out as the controlled condition for comparison purposing. The compounds were then kept and labeled as COMP II. In the final compounding steps, COMPS I and II with controlled Mooney viscosity of 55–60 ML1 + 4 (100 °C) were mixed together in the mixer at 40 °C and 60 rpm for 10 min for proposed ratios of 70:30. The composite were sheeted at 160 °C under the compression molding for getting the crosslinked composites dimensions of 150 × 160 × 2 mm^3^. It is noted that the M-BIIR compounds mixed with ENR-CNT/CB composites for the unfilled and filled TESPT silane coupling agent were indicated the coding as “M-BIIR/ENR-HF” and “M-BIIR/ENR-HF_Si_”, respectively.

## 3. Characterization

### 3.1. Cure Characteristics

Cure characteristics of BIIR/ENR composites with and without TESPT were tested through a moving die rheometer (MDR, Monsanto Co., Ltd., Creve Coeur, MO, USA) under the applied frequency of 1.66 Hz together with 160 °C and 1°. Further, the scorch time (*T_s_*_1_) and the cure time (*T*_90_) which refer to the time required for the 1 dN.m of torque higher than minimal torque (*M_L_*) and for the 90% of chemical crosslinking inside the rubber compound, respectively, and the maximal–minimal torque differences (*M_H_*−*M_L_*) were reported. It is noted that the *T*_90_ is officially used as the production time of rubber composites and products.

### 3.2. Mechanical Properties

Mechanical properties based on tensile testing following the ISO 527 (type 5A) were examined. The tests were performed at room temperature (23 ± 2 °C) under the crosshead speed of 200 mm/min through a tensile testing machine (Zwick Z 1545, Zwick GmbH & Co. KG, Ulm, Germany). For measuring self-heal ability, the dumbbell-shaped samples were also cut with using a sharp razor blade (Energizer^®^ Holdings, Inc., St. Louis, MI, USA) before pressing to the mold. The samples connected with mold were heated at 120 °C for 30 min without applying any pressure. The healed samples were then removed, kept in desiccator for 24 h, and their tensile properties were measured with the same conditioning as the unhealed case. The healing efficiency (X*), which is the divide of the received targeted values at before (*X′*) and after (*X″*) healing conditioning, was then calculated with Equation (1):

(1) $$\;X^{*}\left(\%\right)=\frac{X^{\prime \prime }}{X^{\prime }}\times 100$$

### 3.3. Morphologies

Surface morphologies of the composites filled and unfilled with TESPT were elucidated following the optical microscope (OM) (Carl Zeiss Microscopy GmbH, Oberkochen, Germany). The samples were rapid cut using a razor blade to obtain a smooth surface before measuring. This investigation is to affirm the healing performance of the provided composites.

### 3.4. Attenuated Total Reflection-Fourier Transform Infrared Spectroscopy (ATR-FTIR)

Infrared measurement was used to confirm the chemical reaction among the rubber matrix, filler surface, and silane molecules. The test was fabricated though the ThermoNicolet Avatar 360 FTIR (Thermo Electron Corporation, Madison, WI, USA) following the resolution of 4 cm^−1^ under 64 scans/sample.

### 3.5. Abrasion Resistance

Abrasion of the composites was carried out for indicating the surface detachment resistance during performing in the Taber tester (Model GT-7012-T, GoTech, Taichung, Taiwan). The sample with approximately 110 mm diameter was polished under 72 rpm rolling speed for 1000 rounds and the wear index (*WI*) was calculated with Equation (2):

*WI* (%) = (*W_y_ − W_x_*) × 100/*W_y_* (2)

where *W_x_* and *W_y_* are weight of the sample before and after the test under the controlled condition.

### 3.6. Dynamic Mechanical Properties

Dynamic mechanical performances of the received composites were investigated by using DMA 1 (Mettler-Toledo GmbH., Zurich, Switzerland). The tests were performed in the tension mode with 10 Hz of frequency at 0.2% of strain. The properties were detected in terms of storage modulus, loss modulus, and loss factor (*Tan δ*) under 2 °C/min of heating rate with temperature ranges of −90 to 80 °C.

### 3.7. Electrical Conductivity

Electrical properties were tested using the LCR meter (Hioki IM 3533, Hioki E.E. Corporation, Nagano, Japan) in term of the resistance (*R_P_*) at a frequency of 50 Hz under room temperature condition. The conductivity (*σ*) was obtained following Equation (3):

(3) $$\sigma =\frac{d}{(R_{p})A}$$

where *d* is the sample thickness and *A* assigns to the area of 5 mm electrode.

### 3.8. Filler Flocculation

Filler agglomeration degree of the composites was determined following the Payne effect using a rubber process analyzer (RPA) (Alpha Technologies, Akron, OH, USA) under maximal and minimal strain sweep of 0.56 and 100% at 1 Hz oscillating frequency and 100 °C.

The storage shear moduli (*ε′*) as a function of strain amplitude were reported. The Payne effect value was calculated through the difference of *ε′* at 0.56 and 100%.

## 4. Results and Discussion

### 4.1. Crosslinking Propagation

**Figure 1:**
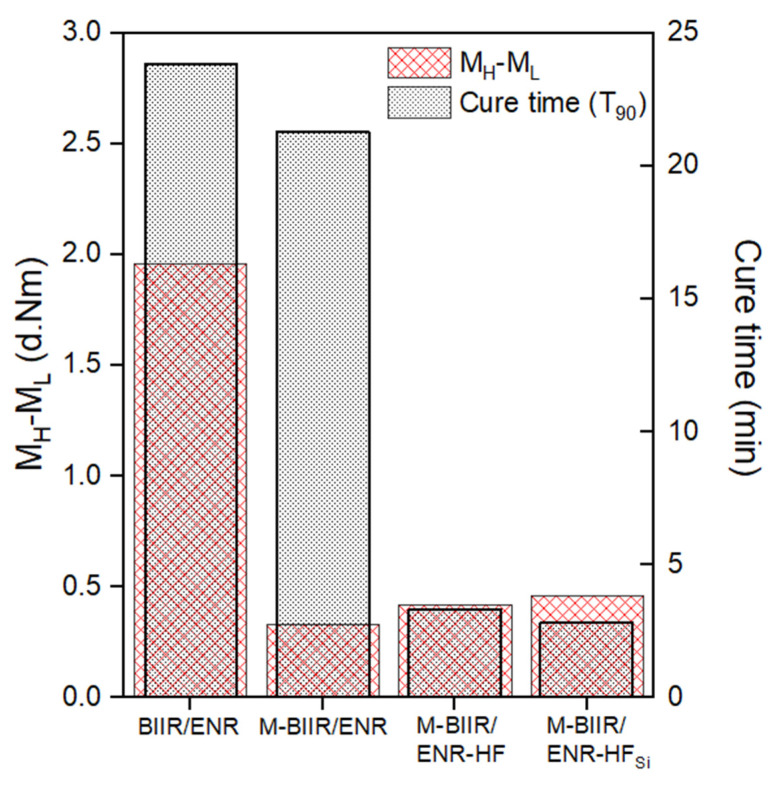
Received of *T*_90_ and *M_H_*−*M_L_* of unmodified BIIR and M-BIIR/ENR composites filled with CNT/CB with and without the addition of TESPT.

Figure 1 shows the cure time (*T*_90_) and torque difference (*M_H_*−*M_L_*) of unmodified BIIR and M-BIIR/ENR composites filled with CNT/CB with and without TESPT to explain the chemical crosslinking propagation of rubber molecules during constant heat as a function of time. Table 2 concludes the curing properties of composites as well as the scorch time (*T_s_*_1_). The *T_s_*_1_ and *T*_90_ strongly decreased with IM and CNT/CB with and without silane. This is owing to the improved dispersion of CNT/CB inside the BIIR/ENR matrix. The higher thermal conductivity of CNT, CB, and IM has been shown to correlate with existing sp^2^-hybridization structures and ionic polarity [15]. Due to the addition of TESPT, CNT and CB agglomeration is broken during mixing by incorporating liquid phases of silane before the salinization reaction when both hybrid filler surfaces are fabricated [13]. The improved dispersion of the carbon-based filler also allows heat to flow simply in the matrix, and the curing fabrication is completed by the rubber molecules and TESPT absorption on the filler surfaces. As the heat expansion increased, the values of *T_s_*_1_ and *T*_90_ decreased [16].

*M_H_*−*M_L_*, which refers to the estimated crosslink density from the vulcanizates, decreased with additional IM but tended to increase after incorporating CNT/CB and CNT/CB with TESPT. The decreasing values of *M_H_−M_L_* confirm that the chemical crosslinking originated in the internal mixture, and the remaining crosslinking was due to sulfur crosslinks between ENR molecules. However, the increased *M_H_−M_L_* after adding CNT/CB with and without TESPT may relate to the chemical linkages between the CNT/CB surface, the TESPT molecules, and the BIIR/ENR chains.

### 4.2. Tensile Properties and Healing Efficiency

**Figure 2:**
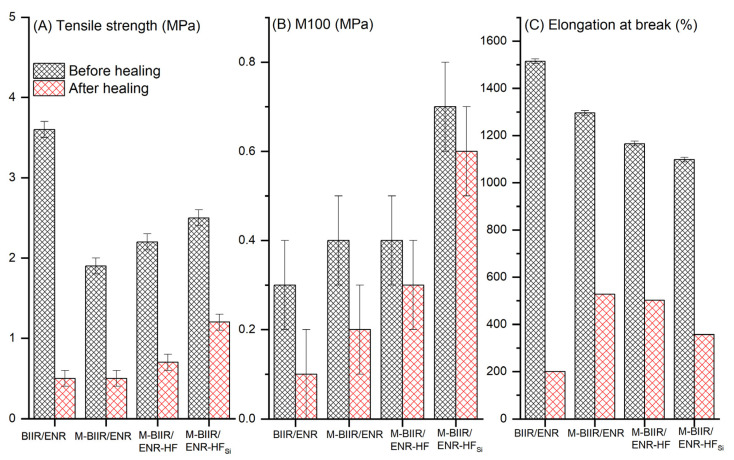
Tensile properties (**A**), modulus at 100% elongation (M100) (**B**) and elongation at break (**C**) of unmodified BIIR and the M-BIIR/ENR composites filled with CNT/CB with and without the addition of TESPT.

The mechanical efficiency of a composite depends on the length of the molecular chains of the rubber, the reinforcement efficiency due to the specific surface area of additional filler, and the filler–rubber interaction [17]. If the fillers are dispersed properly in the chosen rubber matrix, strong rubber–filler interactions are observed because the re-agglomeration after compounding was retarded. In contrast, if the filler–filler interaction is strong, agglomeration can reduce the mechanical properties because of the high number of defects inside the matrix, which easily fail under external forces [18]. Figure 2 shows the tensile properties of M-BIIR vulcanizates mixed with ENR-CNT/CB composites with the unfilled and filled TESPT silane coupling agent. In addition, Table 3 summarizes the modulus at 100% elongation (M100), tensile strength, elongation at break, and Table 4 summarizes of the healing ability of the composites.

In Figure 2A and Table 3, considering the properties before the healing tests, the tensile strength slightly decreased after the modification of BIIR with IM. This is attributed to the different intrinsic bonding strengths of the ionic and *S-S_x_-S* covalent bonds. Here, the latter bond seems to be more flexible, yielding a higher extension ratio. Therefore, the tensile strength and elongation at break (Figure 2B) of the composite cured with the sulfur system alone were higher. In is noted that the two times higher of tensile strength for BIIR/ENR than M-BIIR/ENR might be mainly related to the strain-induced crystallization of ENR molecules chains which had well originated relative to the case of the composite with IM modification, owing to ions–dipole attraction during stretching. However, after adding both hybrid fillers and the silane coupling agent, the tensile strength was higher than either composite without filler.

**Figure 3:**
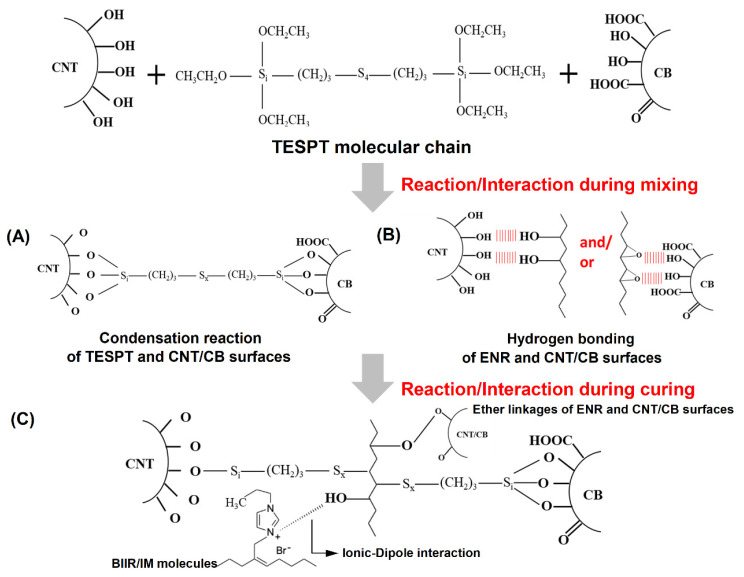
Proposed reaction propagation that occurred during mixing operation of CNT/CCB and TESPT (**A**) and ENR molecular chains (**B**) and during the compression molding by crosslinking with ENR molecules and by both ENR molecules and ion–dipole intermolecular chain (**C**).

The incorporation of CNT/CB caused filler reinforcement in the M-BIIR/ENR matrixes, retarding the composite breakage. In addition, when TESPT was added, the highest tensile strength in the opposite with elongation at break was reported. The addition of TESPT caused a silanization reaction on the CNT/CB surfaces, referred to as the TESPT-grafted CNT/CB, as seen in the proposed model in Figure 3. This reaction propagation occurred after the mixing operation. Then, during the compression process, the other sulfur atomic site in the TESPT was chemically crosslinked to the M-BIIR/ENR molecular chains and the ether linkages among ENR molecular chains and functional groups on CNT/CB surfaces were fabricated (Figure 3C).

It is also seen that the ion–dipole intermolecular chain attraction had faced due to the cations, anions, and hydroxyl groups in the rubber molecules, seen in Figure 3C. Thus, the tensile strength increased because the new chemical linkages restrict the extension of the molecular chains, and the elongation at break was limited after TESPT was added. The large increases in the M100 are shown in Figure 2C and are due to the same reasons. These results were higher than those of the unmodified BIIR/ENR blend. This confirmed the nature of the ionic bonds inside the M-BIIR/ENR, i.e., strong but more brittle than the sulfur bonds.

**Figure 4:**
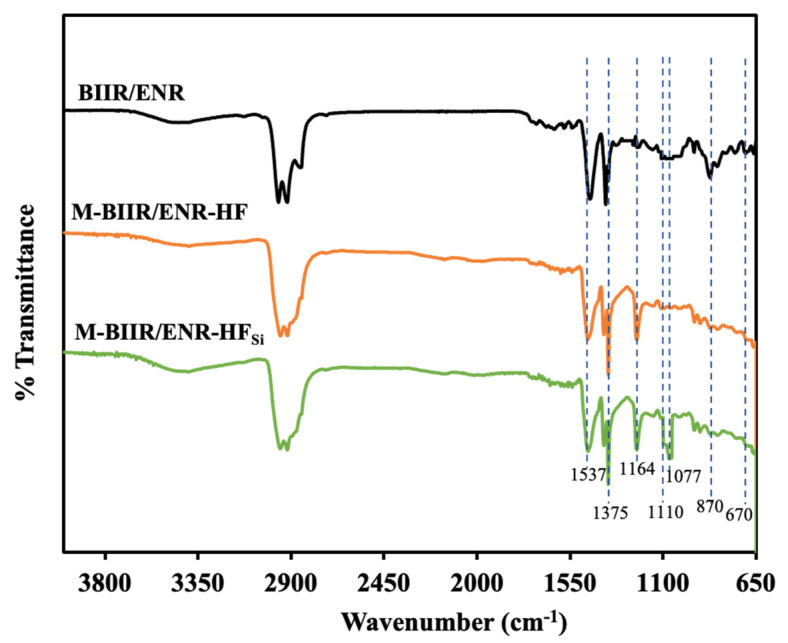
ATR-FTIR of BIIR/ENR of unmodified BIIR and M-BIIR/ENR composites filled with CNT/CB with and without the addition of TESPT.

The modulus increased with added TESPT due to the support of CNT/CB-TESPT/M-BIIR-ENR linkages, which was also confirmed by the attenuated total reflection-Fourier transform infrared (ATR-FTIR) spectra of the composites, as seen in Figure 4 and Table 4. Here, the absorption peaks at 670, 870, and 1164 cm^−1^ were assigned to the –C–Br stretching vibrations from the BIIR chains, the C–O stretching vibrations of the oxirane ring from the ENR chains, and the H–C–N bending vibrations from the IM structures, respectively [19,20]. The peak at 1164 cm^−1^ was observed after the addition of IM, showing that the IM had already grafted at the Br branching position in the BIIR molecular chain for M-BIIR/ENR with and without TESPT. In the case of composites with added silane, the peaks at 1077 and 1100 cm^−1^, which were assigned to the existing Si–O–Si and Si–O–C vibrations, were found [21,22]. In particular, the latter peak indicates that the silanization has already occurred between the TESPT molecules and the CNT/CB surfaces. Thus, these data indicate that the improvement of tensile properties of the composites was due to the addition of the TESPT silane coupling agent [23,24,25].

**Figure 5:**
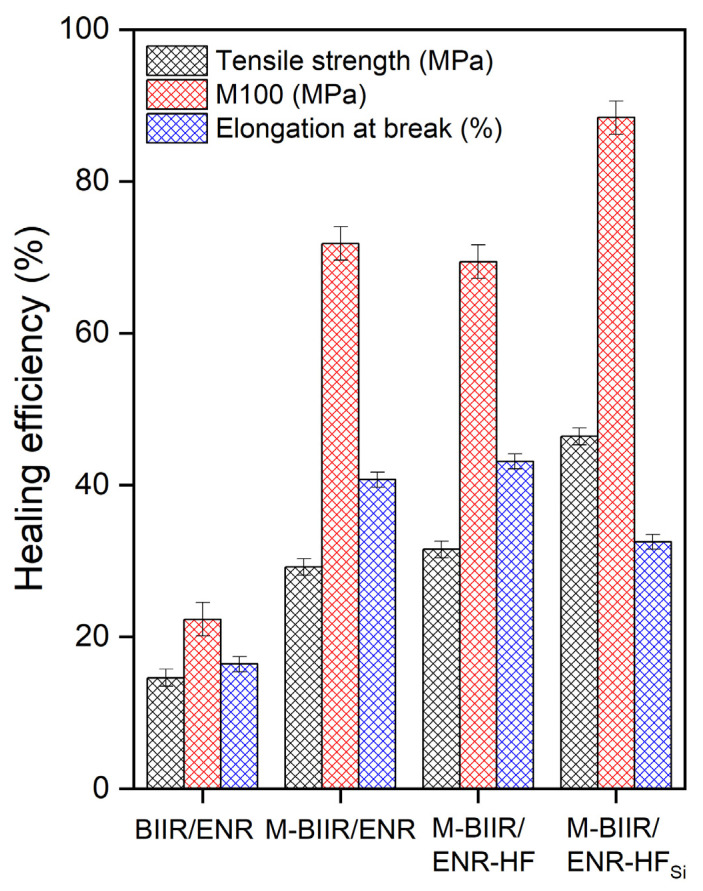
Healing efficiency of unmodified BIIR and the M-BIIR/ENR composites filled with CNT/CB with and without the addition of TESPT based on tensile properties, elongation at break and M100.

Self-healing efficiency is based on the combination of the tensile strength, elongation at break, and M100 of the composite. In Figure 5 and Table 5, modification with BIIR significantly increased the self-re-crosslinking ability of each composite containing additional CNT/CB and CNT/CB with TESPT. This indicates that the ion attraction was well organized even though the hybrid filler was added. The healing of the composites was conditioned in an oven at 120 °C for 30 min without applying pressure. This temperature activated the ionic bonds for re-crosslinking faster than in the composites without modification. For added hybrid filler, high thermal conductivity, as found in the curing characteristics, induced movement of the cations and anions inside the composites, resulting in molecular chains that can re-bond. Thus, if the fillers have good dispersion and distribution throughout the composite matrix, the healing propagation speeds up because the heat expansion is rapidly transferred.

**Figure 6:**
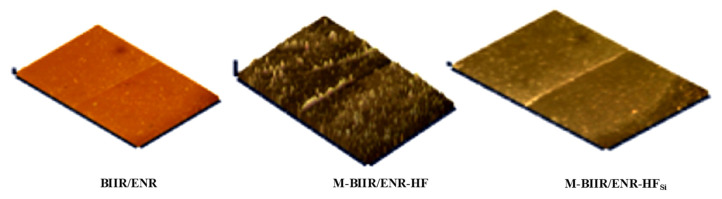
Morphologies of M-BIIR compounds mixed with ENR-CNT/CB composites for the unfilled and filled TESPT silane coupling agent after healing propagation.

In Figure 5, the healing efficiency through the modulus is higher than the other mechanisms. This is attributed to the ionic crosslinks, which are characterized by strong chemical bonding with high brittleness. Thus, at low strains, high stress in the composites was reported. Confirmation of the healing ability of the composites containing M-BIIR is seen in the optical microscopy images in Figure 6. The line in the middle of each sample is the connected surface after 30 min of treatment at 120 °C without pressure. In Figure 6, a rough surface is seen after adding CNT/CB relating the CNT/CB agglomeration inside the matrix [24]. A smoother surface was found in the M-BIIR/ENR-HF_Si_ composite due to the improved dispersion and distribution of the hybrid filler correlated with the addition of the TESPT silane coupling agent. This correlates the work of Nakaramontri et al., which indicated the effect of TESPT silane coupling agent on improvement of CNT/CB dispersion and distribution throughout the rubber matrix [24,25]. Here, the healed surface seems to recover well, effectively supporting the self-healing efficiency results based on the tensile properties of the composites. Considering the BIIR/ENR and M-BIIR/ENR-HF_Si_, the smooth surface of BIIR/ENR is due to there being no CNT/CB hybrid filler inside the composites. The higher healing efficiency of the M-BIIR/ENR-HF_Si_ is related to the strong attraction of the rubber molecular chains absorbed on the filler surface resisting breakage during extension.

### 4.3. Correlated Properties of Abrasion Ability and Dynamic Mechanical Performance

**Figure 7:**
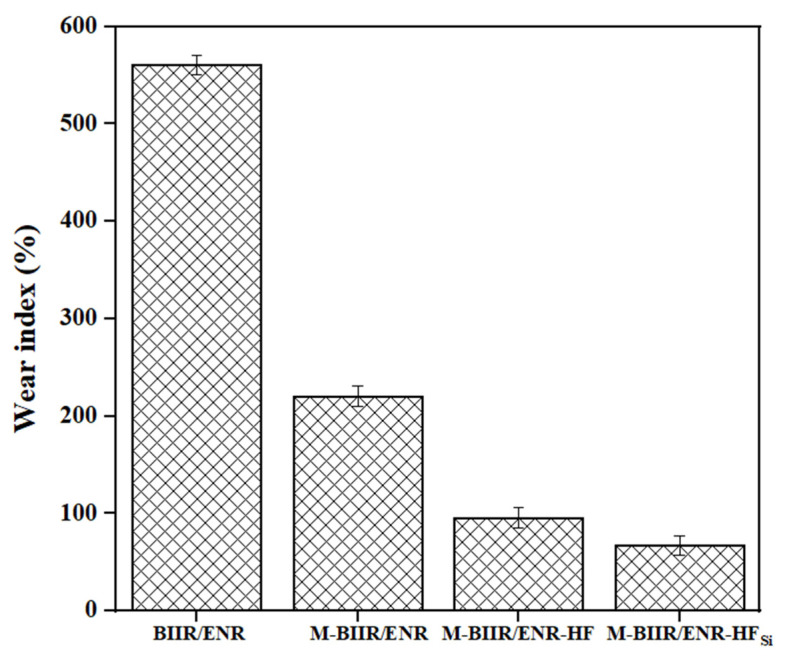
Wear index of unmodified BIIR and the M-BIIR/ENR composites filled with CNT/CB with and without the addition of TESPT.

Figure 7 shows the Taber abrasion results of the composites using the wear index: lower values mean higher wear resistance. The resistance increased after the addition of filler because of the bound rubber. Bound rubber is the absorption of molecular chains of rubber on the filler surfaces, which retards abrasion. The bound rubber limited the movement of the rubber chain out of the surfaces and induced abrasion resistance. In Figure 7, M-BIIR/ENR-HF with and without TESPT have the lowest wear indexes, surpassing the wear index requirement for a range of tires used in passenger cars [16]. The two reasons for high wear resistance are the presence of bound rubber and self-healing ability during abrasion propagation.

**Figure 8:**
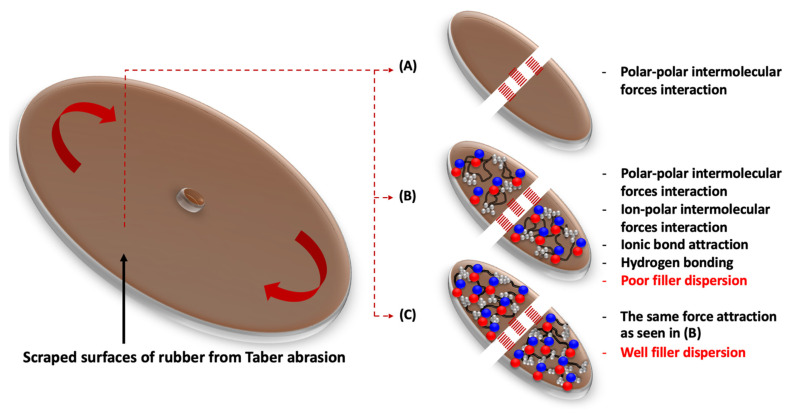
Proposed model of each composite after Taber resistance testing of BIIR/ENR (**A**), M-BIIR/ENR-HF (**B**) and M-BIIR/ENR-HF_Si_ (**C**).

**Figure 9:**
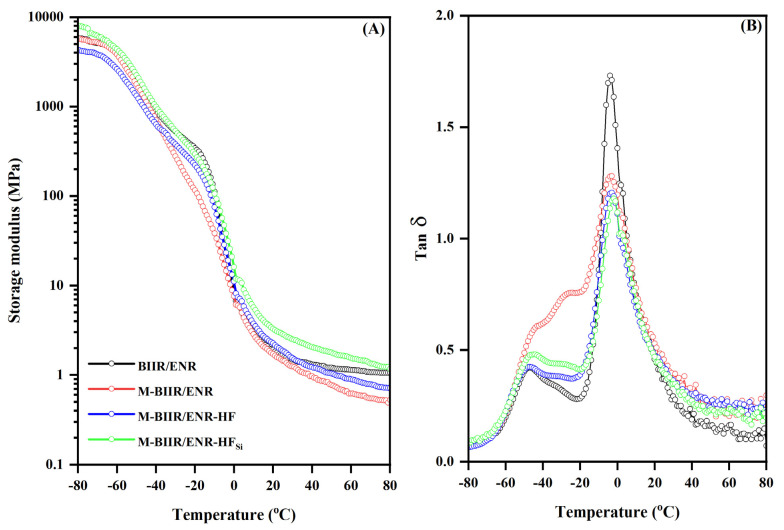
Relationship of the storage modulus (**A**) and *Tan δ* (**B**) with temperature of unmodified BIIR and the M-BIIR/ENR composites filled with CNT/CB with and without the addition of TESPT.

The proposed model for the self-healing ability is shown in Figure 8. When the rubber surfaces were scratched and began to detach, the ionic attraction and ion–dipole forces prevented the failure; therefore, the total rubber loss of the composites was reduced. Further, it must be noted that the existence of the dynamic covalent sulfidic bonds and the epoxy groups’ interaction relating to the uses of the sulfur curing system with additional ENR can be the reasons for proposing the healing propagation. This significantly affected the dynamic mechanical properties of the composites related to elasticity at different temperatures. Figure 9 shows the storage modulus (*E*′) and *tan δ* as a function of temperature. The glass transition temperature (*T_g_*) and *tan δ* at 0 and 60 °C (*tan δ*_0_ and *tan δ*_60_) are summarized in Table 6.

In Figure 9A, the storage modulus curves show the strong influence of the filler on the dynamic behavior of the composites relative to BIIR/ENR and M-BIIR/ENR. In case of composite with TESPT, the highest storage modulus is shown due to well dispersion and distribution of CNT/CB inside the M-BIIR/ENR matrix. This correlates to the M100 value exhibited in Figure 2B. However, for the composite without silane, a strong agglomeration of filler is formed, and a different result is faced relative to the mechanical properties measuring at room temperature. This might be explained by the low temperature during testing in DMA, which caused composites to crack easily under the tension mode relating to strong agglomeration. The reinforcement efficiency can also be elucidated through the rubbery states of Figure 9A for the received storage modulus at a temperature over 0 °C. The highest storage modulus is represented in M-BIIR/ENR-HF_Si_ and this affirms the reinforcement effect of the CNT/CB particles to the M-BIIR/ENR matrix. In addition, regarding to the observed *T_g_* based on *E’* (*T_g_* from *E’*) and *tan δ* (*T_g_* from *tan δ*) in Table 6, it has to be noted that the *T_g_*_1_ and *T_g_*_2_ are related to the *T_g_* of the ENR and BIIR molecular chain, respectively. That is, the modification of BIIR to M-BIIR restricted rubbery chain movement due to the ionic interaction among the molecules, increasing *T_g_* in both BIIR and ENR phases [15]. In addition, the incorporation of CNT/CB caused more restriction of the rubber chains relating to the physical absorption onto filler surfaces. To improve the filler dispersion and distribution, the rubber-absorbed filler surfaces were increased, and this yielded the highest *T_g_* for the M-BIIR/ENR composite filled with CNT/CB and TESPT.

When considering the *tan δ*_0_ and *tan δ*_60_ obtained in this study, it is noted that in tire applications, the *tan δ*_0_ that is reported is the prediction of the wet traction, while *tan δ*_60_ represents the energy loss of the composites due to the dynamic deformation associated with the rolling resistance [19]. Thus, a low *tan δ*_60_ paired with a high *tan δ*_0_ is required for producing self-healing tire application, whereas a low *tan δ* value indicates high elasticity. In Figure 9B and Table 6, *tan δ*_60_ strongly increases after modification, and it tends to decrease after the addition of CNT/CB with and without TESPT. Because *tan δ* is the ratio of loss to storage moduli, decreased *tan δ*_60_ indicates increased storage modulus or decreased of loss modulus; therefore, the rolling resistance of the composite is lowered after the addition of TESPT—an important factor for tires at high temperatures.

In contrast, the opposite results were observed for *tan δ*_0_. At 0 °C, high recovery of the composites after physical deformation related to the ion attraction. This elasticity decreased after the addition of CNT/CB and CNT/CB with TESPT because *tan δ*_0_ increased. This reflects improved wet traction, which improves driving on the targeted wet and cold material surfaces. The improved self-healing efficiency due to the addition of TESPT also provided proper performances at the required high and low-temperature conditions.

### 4.4. Electrical Conductivity

**Figure 10:**
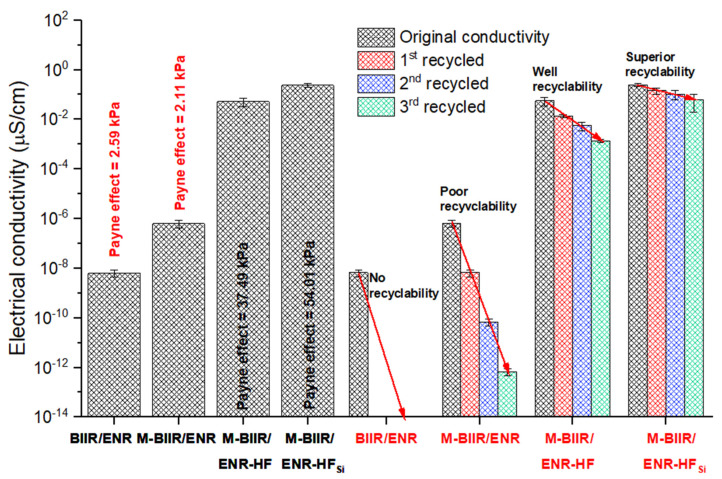
Electrical conductivity of unmodified BIIR and the M-BIIR/ENR composites filled with CNT/CB with and without the addition of TESPT.

The improved electrical conductivity of the CNT/CB network formed inside the M-BIIR/ENR matrixes is explained by the transfer of electrons throughout the hybrid filler, as seen in Figure 10. The slight increase in the conductivity observed after the modification was due to the enhanced electron movement in the composites. In addition, an increase of approximately 10 orders of magnitude in the conductivity was observed, and the composite changed from an insulator to a semiconductor. The electrical conductivity again increased tenfold after the incorporation of TESPT due to the improved CNT/CB hybrid filler dispersion and distribution. The CNT/CB formed a good network inside the rubber matrix. To confirm the filler networks, the Payne effect was determined based on the different storage moduli at the lowest and highest strain amplitudes. A high Payne effect indicates high amounts of contact between the filler particles, which signals good dispersion of the CNT fibers, as seen in the inset of Figure 10. This correlates well to the indication of composites surface indicating in Figure 6 based on OM measurement.

Self-healing rubber composites not only have rubber repairing and high abrasion resistance but can also improve reusability. Figure 10 also shows the electrical conductivity of the composites filled with CNT/CB and CNT/CB-TESPT after two and three re-compressions. Here, the rubber vulcanizate sheets are cut into approximately 1.0 × 1.0 × 0.2 cm^3^ before re-compression to make sheets. Both composites had good recyclability due to the conductivity recovered after recycling. In addition, the composites that incorporated TESPT showed a higher degree of conductivity due to the dispersion and distribution of the hybrid filler inside the rubber matrix. Therefore, the self-healing composites can be reused and showed the same electrical conductivity due to the rearrangement of the CNT/CB filler pathways, although the vulcanizates were re-sheeted.

## 5. Conclusions

Self-healing composites were successfully manufactured based on BIIR/ENR and M-BIIR/ENR-HF with and without the TESPT silane coupling agent. The addition of CNT/CB hybrid filler with and without TESPT significantly improved the curing characteristics by shortening the production time of the composites relative to the BIIR/ENR and M-BIIR/ENR blends. The incorporation of TESPT provided increased tensile properties in terms of tensile strength and modulus, although elongation at break tended to decrease because of the restricted movement of the molecular chains of rubber. New chemical linkages from the added silane effectively increased the self-healing efficiency. This is an opportunity for several applications, particularly tire technology. Therefore, the Taber abrasion was measured, and the lowest value was observed for the composite filled with silane, as expected. This phenomenon is controlled by decreasing the detachment of rubber particles during abrasive application due to ion–ion and ion–dipole attraction.

Furthermore, the M-BIIR/ENR composite filled with CNT/CB and TESPT exhibited excellent usability at temperatures of 0 and 60 °C, which correspond to the lowest and highest *tan δ*_60_ and *tan δ*_0_ values relative to the M-BIIR/ENR and M-BIIR/ENR-HF. In addition, the recyclability of the composites was established using the observed electrical conductivity based on original and recompressed samples. These composites can support future applications that require self-repairing capabilities, simple preparation and short production processes, better semiconductor solidification dispersion, recyclability, high durability in terms of wear resistance, and variable material flexibility at low and high operating temperatures.

## Figures and Tables

**Table 1:** Formulation design of the pure rubber and their composites.

Chemicals	Contents (phr *)
**COMP I**	
BIIR	100.0
IM	5.0
**COMP II**	
ENR	100.0
CNT:CB	5.0:7.5
ZnO	5.0
Stearic acid	1.0
MBTS	1.0
Sulfur	2.5
TESPT	Fix concentration **

* = part per hundred rubber, ** = 0.06 g/mL [13].

**Table 2:** Details of chemical crosslink propagation extracted from the cure characteristic curves indicated in Figure 1.

Samples	*T*_90_	*T_s_*_1_	*M_H_*−*M_L_*
BIIR/ENR	23.85	19.07	1.96
M-BIIR/ENR	21.26	8.75	0.33
M-BIIR/ENR-HF	3.28	4.25	0.42
M-BIIR/ENR-HF_Si_	2.82	2.25	0.46

**Table 3:** Mechanical properties and healing efficiency of M-BIIR compounds mixed with ENR-CNT/CB composites for the unfilled and filled TESPT silane coupling agent.

Sample	Tensile Strength (MPa)		Elongation at Break (%)		M100 (MPa)	
	Before Healing	After Healing	Before Healing	After Healing	Before Healing	After Healing
BIIR/ENR	3.6 ± 0.1	0.5 ± 0.1	1515.0 ± 9.8	200.0 ± 0.1	0.3 ± 0.1	0.1 ± 0.0
M-BIIR/ENR	1.9 ± 0.1	0.5 ± 0.1	1296.0 ± 10.1	528.0 ± 0.1	0.4 ± 0.1	0.2 ± 0.1
M-BIIR/ENR-HF	2.2 ± 0.1	0.7 ± 0.1	1166.1 ± 9.9	502.0 ± 0.1	0.4 ± 0.1	0.3 ± 0.1
M-BIIR/ENR-HF_Si_	2.5 ± 0.1	1.2 ± 0.1	1098.1 ± 10.1	357.0 ± 0.1	0.7 ± 0.1	0.6 ± 0.1

**Table 4:** Assignments of the peak of infrared spectra in Figure 4.

Wavenumber (cm^−1^)	Assignments
670	C–Br stretching vibrations
870	C–O stretching vibrations of oxirane ring
1077	Si–O-Si vibrations
1110	Si–O–C Vibrations
1164	H–C–N bending vibrations
1357	Asymmetric and symmetric C–H stretching vibrations
1537	C = O stretching vibration of Zinc stearate

**Table 5:** Healing efficiency based on tensile properties, elongation at break and M100.

Samples	Healing Efficiency (%)		
	Tensile Strength	Elongation at Break	M100
BIIR/ENR	14.6 ± 1.1	16.4 ± 1.0	22.3 ± 2.2
M-BIIR/ENR	29.2 ± 1.1	40.7 ± 1.0	71.8 ± 2.2
M-BIIR/ENR-HF	31.5 ± 1.1	43.1 ± 1.0	69.4 ± 2.2
M-BIIR/ENR-HF_Si_	46.4 ± 1.1	32.5 ± 1.0	88.4 ± 2.2

**Table 6:** Dynamic mechanical of M-BIIR compounds mixed with ENR-CNT/CB composites for the unfilled and filled TESPT silane coupling agent.

Sample	*T_g_* from *E′*		*T_g_* from *Tan δ*		*Tan δ* at 60 °C	*Tan δ* at 0 °C
	*T_g_*_1_	*T_g_*_2_	*T_g_*_1_	*T_g_*_2_		
BIIR/ENR	−54	−12	−47	−4	0.16	1.41
M-BIIR/ENR	−51	−11	−44	−3	0.26	1.12
M-BIIR/ENR-HF	−49	−11	−41	−3	0.25	1.13
M-BIIR/ENR-HF_Si_	−47	−10	−38	−2	0.20	1.20

## Data Availability

The data presented in this study are available on request from the corresponding author.
